# Efficacy and Safety of Drug-Eluting Beads Transarterial Chemoembolization Combining Immune Checkpoint Inhibitors in Unresectable Intrahepatic Cholangiocarcinoma: A Propensity Score Matching Analysis

**DOI:** 10.3389/fimmu.2022.940009

**Published:** 2022-07-08

**Authors:** Xue-Gang Yang, Yan-Yuan Sun, De-Shan Li, Guo-Hui Xu, Xiao-Qi Huang

**Affiliations:** ^1^ Huaxi MR Research Center (HMRRC), Functional and Molecular Imaging Key Laboratory of Sichuan Province, Department of Radiology, West China Hospital, Sichuan University, Chengdu, China; ^2^ Department of Interventional Radiology, Sichuan Cancer Hospital and Institute, Chengdu, China

**Keywords:** unresectable intrahepatic cholangiocarcinoma, transarterial chemoembolization, immune checkpoint inhibitor, chemotherapy, combined therapy

## Abstract

**Purpose:**

To assess the effectiveness and safety of drug-eluting beads transarterial chemoembolization plus immune checkpoint inhibitors (DEB-TACE+ICIs) versus chemotherapy (gemcitabine+cisplatin) for patients with unresectable intrahepatic cholangiocarcinoma (iCCA).

**Materials and Methods:**

This retrospective study included unresectable iCCA patients treated with DEB-TACE+ICIs or chemotherapy between May, 2019 and August, 2021. The differences in tumor responses, progression-free survival (PFS), overall survival (OS), and treatment-related adverse events (TRAEs) were compared between the 2 groups. Patient baseline characteristics, PFS, and OS were compared among 2 groups before and after propensity score-matching (PSM). Factors affecting PFS and OS were analyzed by Cox’s proportional hazards regression model.

**Results:**

The study included 49 patients with unresectable iCCA patients, 20 in the DEB-TACE+ICIs group and 29 in the chemotherapy group. PSM analysis created 20 pairs of patients in 2 groups. The patients in the DEB-TACE+ICIs group had a higher objective response rate (55.0% vs. 20.0%, *P*=0.022), higher PFS (median, 7.2 vs. 5.7 months, *P*=0.036), and higher OS (median, 13.2 vs. 7.6 months, *P*=0.015) than those in the chemotherapy group. Multivariate analyses suggested that chemotherapy, tumor size >5cm, and multiple tumors were the independent risk factors for PFS and OS. The incidence of TRAEs was similar between the 2 groups.

**Conclusion:**

Compared to chemotherapy, DEB-TACE plus ICIs improved survival and was well-tolerated in patients with unresectable iCCA.

## Introduction

Intrahepatic cholangiocarcinoma (iCCA) is the second most common primary liver malignancy. In the last decade, its global incidence has increased from 0.44 per, 100000 to 1.18 cases per, 100000; the mortality has increased from 1.5 per, 100000 to 2.5 cases per, 100000 in men and 1.2 per, 100000 to 1.7 cases per, 100000 in women (1, 2). The iCCA patients are often asymptomatic, the disease is usually accidentally discovered, typically by imaging, when in an advanced stage; thus, most patients have a poor prognosis. The major clinical symptoms are abdominal pain, jaundice, and weight loss (3). Considering the advanced disease stage, including vascular invasiveness and distal metastasis, most iCCA (approximately 80%) patients lose their chance to undergo surgical resection and transplantation (4, 5). Moreover, even after treatment, early recurrence and metastasis are prone to occur.

The median overall survival (OS) of untreated iCCA patients has been reported to be 3 to 6 months (6, 7). Previous study has suggested that chemotherapy (gemcitabine+cisplatin) can improve the clinical outcomes for unresectable iCCA (8). However, many patients have a chemo-refractory or discontinue chemotherapy due to severe adverse reactions associated with treatment. Thus, new treatment methods have been proposed, including loco-regional therapy, biological therapy, and targeted therapy (3, 9); yet, there is still no consensus on the best therapy for unresectable iCCA.

Over the years, there has been much interest in transarterial chemoembolization (TACE) (10). However, minimal vascularity and lower drug concentration due to leakage of loading chemotherapeutic agents are the biggest challenges when treating patients with iCCA (11). Recent studies have found that drug-eluting beads transarterial chemoembolization (DEB-TACE) can enhance intratumoral drug penetration and reduce systemic side effects compared to conventional TACE (12, 13). Moreover, the OS of unresectable iCCA patients treated with DEB-TACE was 9-10 months (14–16).

Rapid advances in cancer immunotherapy using immune checkpoint inhibitors (ICIs), such as atezolizumab combined with bevacizumab, have significantly improved outcomes in unresectable hepatocellular carcinoma (HCC) compared to sorafenib (17). Recently, China Food and Drug Administration (CFDA) approved camrelizumab combined with chemotherapy (gemcitabine+cisplatin) (Gemox) as a first-line systemic treatment for advanced biliary tract cancer based on phase II study results (18), and sintilimab plus bevacizumab biosimilar (Ibi305) as a first-line systemic therapy for middle-advanced HCC based on phase II/III study results (19).

DEB-TACE is used to achieve more extensive tumor necrosis, which can induce anti-tumor immune response in patients with unresectable iCCA; yet, it may not confer long-time anti-tumor effect. However, combining DEB-TACE with ICIs may further increase the development of tumor antigen-specific memory T cells, sustaining anti-tumor responses in unresectable iCCA patients (20). Thus, the aim of current study was to examine and compare the efficacy and safety of DEB-TACE combined with immune checkpoint inhibitors (DEB-TACE+ICIs) versus chemotherapy (gemcitabine+cisplatin) for unresectable iCCA patients.

## Materials and Methods

### Patient Cohort

This retrospective study was performed in accordance with the principles of the Declaration of Helsinki and was approved by the ethical review committee of Sichuan Cancer Hospital. The requirement to obtain informed patient consent was waived. Clinical data of patients with unresectable iCCA who underwent DEB-TACE+ICIs or chemotherapy as first-line therapy at Sichuan Cancer Hospital between May, 2019 and August, 2021 were analyzed. Unresectable iCCA included multifocal tumors, extensive regional lymphadenopathy, distant metastases, non-reconstructable vascular involvement, or severe underlying liver parenchymal disease. Patients were initially treated with DEB-TACE plus ICIs because of the rejection of system chemotherapy.

The inclusion criteria were: 1) age between 18 and 80 years; 2) histologically or cytologically confirmed diagnosis of iCCA; 3) Eastern Cooperative Oncology Group performance score (ECOG PS) of ≤ 2; 4) Child-Pugh class ≤ 7. The exclusion criteria were: 1) with other malignancies; 2) previously received TACE, curative resection, ablation, hepatic arterial infusion chemotherapy (HAIC), other systemic treatment, or radiotherapy; 3) current or previous central nervous system metastasis; 4) mixed feature iCCA-HCC; 5) with current or previous severe cardiovascular disease or coagulation disorders; 6) incomplete clinical or imaging data.

### Preoperative Evaluation

For research analysis, we collected the preoperative clinical data from the medical record systems: sex, age, ECOG PS, Child-Pugh class, carcinoembryonic antigen (CEA), carbohydrate antigen 199 (CA_199), tumor number, tumor size, extrahepatic metastasis, hematologic and biochemical indexes. Also, all patients underwent contrast-enhanced magnetic resonance imaging (MRI) or computed tomography (CT) scan one week before the first treatment.

### Chemoembolization Procedure

CalliSpheres® (Jiangsu Hengrui Medicine Co. Ltd, Jiangsu, China) beads (100-300 μm) were loaded with doxorubicin (50 - 80 mg). The loading process (21) was: 1) the concentration of doxorubicin was 20 mg/ml; 2) the supernatant of CalliSpheres® beads was excluded, then beads and doxorubicin were mixed; 3) non-ionic contrast agent was added into the mixture (using a 1:1 ratio) for further application.

Before performing chemoembolization, celiac arteriography and superior mesenteric arteriography were implemented to evaluate the feeding arteries of the tumor. Then, microcatheters were used to catheterize the tumor-feeding arteries. The mixture of CalliSpheres® beads and non-ionic contrast agent were injected at the speed of 1 ml/min. The injection was completed if the stasis flow of the contrast agent was observed. If one vial of CalliSpheres® beads did not complete the chemoembolization, regular Embosphere (Biosphere Medical, Roissy en France, France) with 100-300 μm was used.

DEB-TACE was repeated “on demand” in patients with no deteriorating physical status or organ function after contrast-enhanced MRI or CT detected viable tumors during follow-up.

### Immune Check Inhibitors (ICIs) and Chemotherapy Administration

Administration of ICIs was first within one week after the initiated DEB-TACE. Intravenous administration of 200 mg camrelizumab (Hengrui Medical, Suzhou, China) or sintilimab (Innovent Biologics, Suzhou, China) was conducted every 3 weeks; the administration was stopped if severe toxicity, tumor progression, or death appeared. Dose interruption, but not reduction, was allowed.

In the chemotherapy group, intravenous administration of every cycle comprised cisplatin (25mg per square meter) followed by gemcitabine (1000 mg per square meter); every drug was performed on day 1 and 8 every 21 days, first for four cycles. If there was no tumor progression at four cycles, patients could continue treatment for another 12 weeks using the same regimen. However, if patients could not tolerate the adverse reaction to therapy, discontinuation or reduction of the dose was recommended and determined by oncologists with more than 10 years of experience in the field.

### Postoperative Follow-Up

All patients were regularly follow-up at intervals of 3-6 weeks after the first treatment. The follow-up included physical examination, contrast-enhanced MRI or CT of the abdomen, chest CT, laboratory tests, and other examinations. The last follow-up time was January 31, 2022.

During follow-up, DEB-TACE+ICIs or chemotherapy was stopped when intolerable toxicity, disease progression, or change in the treatment regimen occurred. The choice of follow-up treatment, such as a change in chemotherapy regimen, ICIs (chemotherapy group), radiotherapy, ablation, TACE (chemotherapy group), or optimal supportive care, was performed based on discussions between our multidisciplinary liver tumor team and the patient’s requirements.

### Assessments

Tumor responses were assessed by 2 radiologists with 10 years of experience based on the modified Response Evaluation Criteria in Solid Tumors (mRECIST). Multiple tumors were defined as more than one tumor. Tumor responses were categorized as complete response (CR), partial response (PR), stable disease (SD), or progression disease (PD). The objective response rate (ORR) was defined as the proportion of patients achieving CR and PR. Disease control rate (DCR) was defined as the proportion of patients with complete, partial response, or stable disease. Tumor responses of all patients were confirmed no less than four weeks after the initial observation.

Progression-free survival (PFS) was defined as the time from the first day of inpatients to PD or death from any cause, whichever occurred first. OS was defined as the time from to the first day of inpatients to the time of death or the last follow-up date.

Adverse events (AEs) were recorded and evaluated based on the Common Terminology Criteria for Adverse Events Version 5.0.

### Statistical Methods

Statistical analyses were carried out using the SPSS Statistics 25.0 (SPSS Inc., Chicago, IL, USA). The propensity score model included age, sex, tumor number (single or multiple), and tumor differentiation. The model provided a 1:1 match between the 2 groups, as previously described (22). Before and after propensity score matching (PSM), categorical data were expressed as frequency; quantitative data were expressed as mean ± standard deviation and median (range) for normally and non-normally distributed variables, respectively. To determine the significant differences between the 2 groups, continuity correction and Mann-Whitney U test, Chi-square, or Fisher exact test were used. Survival curves of PFS and OS were analyzed by the Kaplan–Meier method using the log-rank test. Univariate and multivariate analyses were used Cox’s proportional hazards regression model to determine the prognostic factors. Variables (*P<*0.1) in the univariate analysis were entered into the multivariable analysis to look for predictors of efficacy. A two-sided *P* level less 0.05 was considered statistically significant.

## Results

### Patient Characteristics

**Figure 1:**
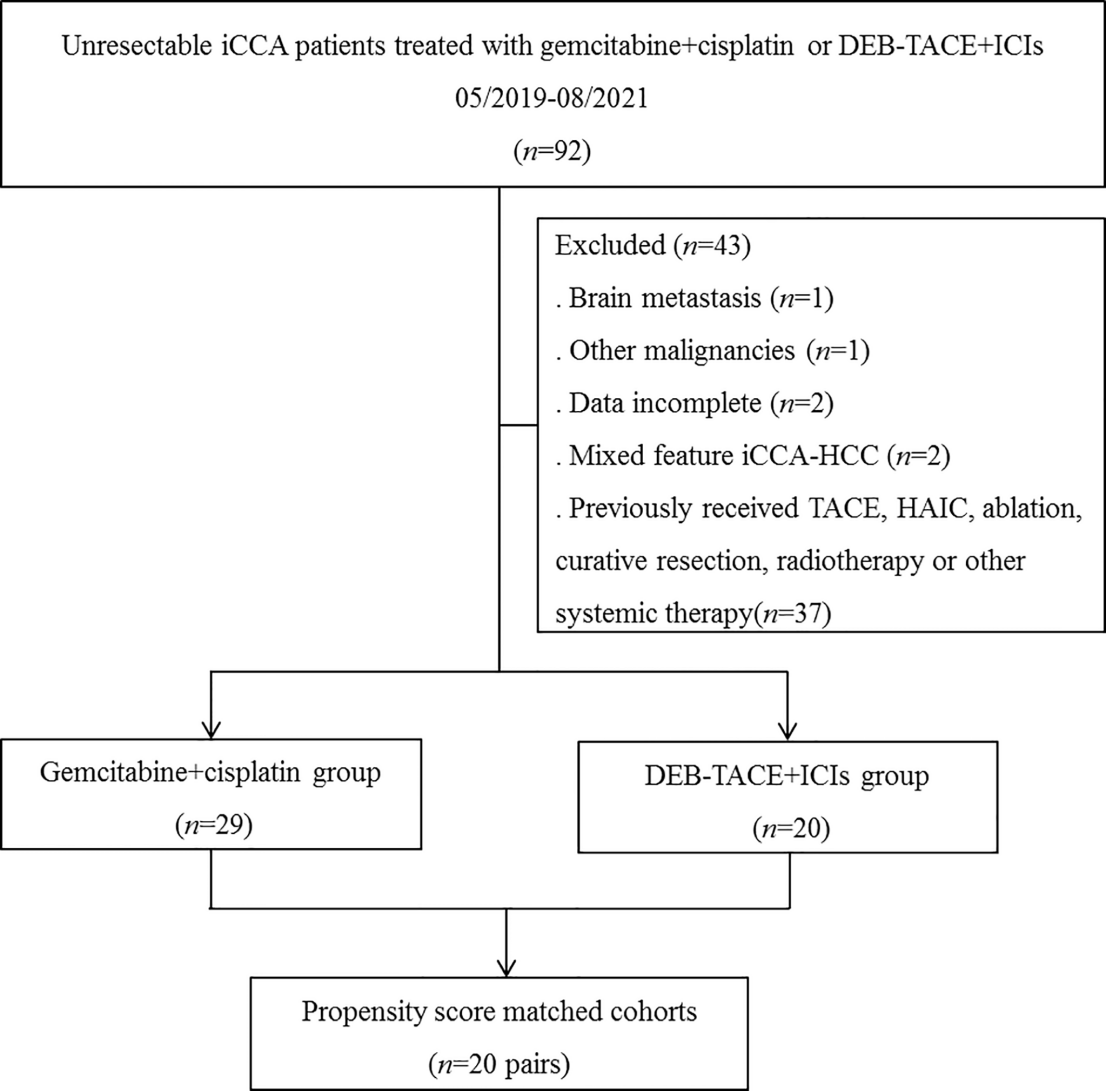
Flow diagram of patient enrollment. iCCA, intrahepatic cholangiocarcinoma; DEB-TACE+ICIs, drug-eluting beads transarterial chemoembolization combined with immune checkpoint inhibitors; TACE, transarterial chemoembolization; HAIC, hepatic arterial infusion chemotherapy.

Totally 49 patients with unresectable iCCA were included in current study, 20 patients received DEB-TACE+ICIs and 29 patients received chemotherapy (**Figure 1**). In the DEB-TACE+ICIs group, 7 patients received camrelizumab, and 13 patients received sintilimab.

PD was seen in 2 patients after DEB-TACE+ICIs treatment; 1 patient received chemoradiotherapy and another patient received systemic chemotherapy. In addition, PD was observed in 5 patients after chemotherapy; 2 patients received a change of chemotherapy regimen, 1 patient received DEB-TACE+ICIs, and 2 patients received chemoradiotherapy.

Before PSM, patients in the chemotherapy group had a higher aspartate aminotransferase (AST)(*P*=0.035) and albumin (*P*=0.018) compared to those in the DEB-TACE group (**Table 1**). Performing PSM resulted in matched cohorts of 20 patients every group with well-balanced baseline characteristics (**Table 1**).

**Table 1:** Baseline characteristics of patients in the two groups before and after PSM.

	Before PSM			After PSM		
Characteristics, n (%)	DEB-TACE+ICIs (n=20)	Chemotherapy (n=29)	*P*	DEB-TAEC+ICIs (n=20)	Chemotherapy (n=20)	*P*
Median age, years (range)	59 (34-76)	59 (31-78)	0.906	59 (34-76)	59 (31-74)	>0.999
≤ 60	10 (50.0)	15 (51.7)		10 (50.0)	10 (50.0)	
> 60	10 (50.0)	14 (48.3)		10 (50.0)	10 (50.0)	
Sex			0.458			0.519
Male	11 (55.0)	19 (65.5)		11 (55.0)	13 (65.0)	
Female	9 (45.0)	10 (34.5)		9 (45.0)	7 (35.0)	
ECOG PS			0.353			0.429
0	8 (40.0)	8 (27.6)		8 (40.0)	5 (25.0)	
1	10 (50.0)	20 (69.0)		10 (50.0)	14 (70.0)	
2	2 (10.0)	1 (3.4)		2 (10.0)	1 (5.0)	
Child-Pugh class			0.388			0.348
A5	13 (65.0)	22 (75.9)		13 (65.0)	16 (80.0)	
A6	3 (15.0)	5 (17.2)		3 (15.0)	3 (15.0)	
B7	4 (20.0)	2 (6.9)		4 (20.0)	1 (5.0)	
CA199, U/ml			0.938			0.723
≤ 37	6 (30.0)	9 (31.0)		6 (30.0)	5 (25.0)	
> 37	14 (70.0)	20 (69.0)		14 (70.0)	15 (75.0)	
CEA, ng/ml			0.062			0.197
≤ 5	10 (50.0)	7 (24.1)		10 (50.0)	6 (30.0)	
> 5	10 (50.0)	22 (75.9)		10 (50.0)	14 (70.0)	
AST, U/L	32.3±16.3	58.1±54.2	0.035	32.3±16.3	38.1±17.4	0.377
ALT, U/L	28 (12-75)	40 (15-175)	0.080	28 (12-75)	39.5 (19-175)	0.137
Albumin, g/dl	38.8±5.5	39.4±3.4	0.018	38.8±5.5	38.6±3.7	0.270
Bilirubin (μmol/L)	11.9 (6.8-36.1)	14.1 (4.7-95.9)	0.143	11.9 (6.8-36.1)	12.5 (4.7-95.9)	0.315
WBC (x10^9^/L)	7.1±1.6	6.3±2.0	0.817	7.1±1.6	6.9±2.0	0.640
Neutrophile (x10^9^/L)	5.1±1.4	4.4±1.7	0.940	5.1±1.4	4.9±1.7	0.864
PLT (x10^9^/L)	196±75	187±61	0.610	196±75	200±58	0.472
HGB (g/L)	125±18	129±18	0.870	125±18	132±17	0.897
Tumor number			0.923			0.206
Single	12 (60.0)	17 (58.6)		12 (60.0)	8 (40.0)	
Multiple	8 (40.0)	12 (41.4)		8 (40.0)	12 (60.0)	
Tumor size, cm	6.7±3.0	5.8±3.0	0.544	6.7±3.0	6.3±2.9	0.889
≤ 5	6 (30.0)	14 (48.3)		6 (30.0)	8 (40.0)	
> 5	14 (70.0)	15 (51.7)		14 (70.0)	12 (60.0)	
Tumor differentiation			0.592			0.765
II	5 (25.0)	7 (24.1)		5 (25.0)	5 (25.0)	
III	7 (35.0)	14 (48.3)		7 (35.0)	9 (45.0)	
IV	8 (40.0)	8 (27.6)		8 (40.0)	6 (30.0)	
Extrahepatic metastasis			0.945			>0.999
Yes	15 (75.0)	22 (75.9)		15 (75.0)	15 (75.0)	
No	5 (25.0)	7 (24.1)		5 (25.0)	5 (25.0)	

PSM, propensity score matching; DEB-TACE+ICIs, drug-eluting bead transarterial chemoembolization combined with immune checkpoint inhibitors; ECOG PS, Eastern Cooperative Oncology Group performance score; CA199, Carbohydrate antigen_199; AST, aspartate aminotransferase; CEA, carcinoembryonic antigen; ALT, alanine transaminase; WBC, white blood cell; PLT, platelet; HGB, Hemoglobin.

### Tumor Response Evaluation

The PR and ORR were higher in the DEB-TACE+ICIs group than those in the chemotherapy group before PSM (PR, 50.0% vs. 13.8%, respectively, *P*=0.006; ORR, 55.0% vs. 13.8%, respectively, *P*=0.002) and after PSM (PR, 50.0% vs. 20.0%, respectively, *P*=0.047; ORR, 55.0% vs. 20.0%, respectively, *P*=0.022), and the DCR of 2 groups before and after PSM were similar (respectively, *P*>0.05)(**Table 2**).

**Table 2:** Summary of response rates before and after PSM.

All response, n (%)	Before PSM				After PSM		
	DEB-TACE+ICIs (n=20)	Chemotherapy (n=29)	*P*	DEB-TACE+ICIs (n=20)		Chemotherapy (n=20)	*P*
CR	1(5.0)	0 (0)	0.224	1 (5.0)		0 (0)	0.311
PR	10 (50.0)	4 (13.8)	0.006	10 (50.0)		4 (20.0)	0.047
SD	7 (35.0)	20 (69.0)	0.019	7 (35.0)		13 (65.0)	0.058
PD	2 (10.0)	5 (17.2)	0.476	2 (10.0)		3 (15.0)	0.633
ORR DCR	11 (55.0) 18 (90.0)	4 (13.8) 24 (82.8)	0.002 0.476	11 (55.0) 18 (90.0)		4 (20.0) 17 (85.0)	0.022 0.633

PSM, propensity score matching; DEB-TACE+ICIs, drug-eluting bead transarterial chemoembolization combined with immune checkpoint inhibitors; CR, complete response; PR, partial response; SD, stable disease; PD, progressive disease; ORR, objective response rate; DCR, disease control rate.

### Survival Analysis

The median follow-up time was 7.2 months (range, 2.8-28.5 months) in this study. In addition, 55.0% (11/20) patients in the DEB-TACE group and 69% (20/29) patients in the chemotherapy group died.

**Figure 2:**
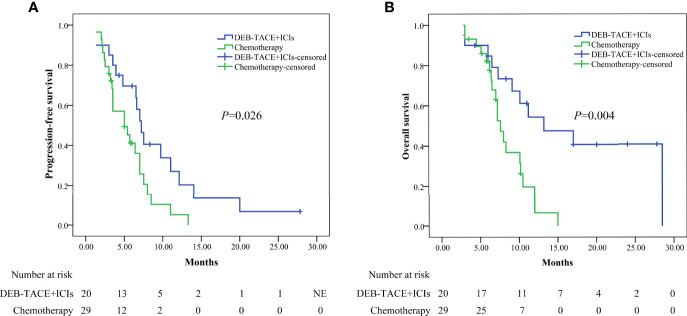
Kaplan-Meier analyses of progression-free survival **(A)** and overall survival **(B)** in the two groups before PSM. DEB-TACE+ICIs, drug-eluting beads transarterial chemoembolization combined with immune checkpoint inhibitors; PSM, propensity score matching.

Before PSM, the median PFS was higher in the DEB-TACE+ICIs group than in the chemotherapy group: 7.2 months (95% CI: 6.012–8.388) versus 5.0 months (95% CI: 2.390–7.610)(*P*=0.026, **Figure 2a**); the median OS was higher in the DEB-TACE+ICIs group than in the chemotherapy group: 13.2 months (95%: CI 4.977–21.423) versus 7.6 months (95% CI: 6.583–8.617) (***P*** = 0.004, **Figure 2b**).

**Figure 3:**
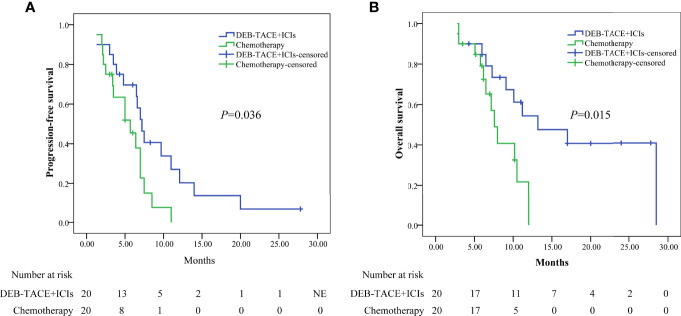
Kaplan-Meier analyses of progression-free survival **(A)** and overall survival **(B)** in the two groups after PSM. DEB-TACE+ICIs, drug-eluting beads transarterial chemoembolization combined with immune checkpoint inhibitors; PSM, propensity score matching.

Performing PSM, the median PFS was higher in the DEB-TACE+ICIs group than in the chemotherapy group: 7.2 months (95% CI: 6.012–8.388) versus 5.7 months (95% CI: 3.056–8.344)(*P*=0.036, **Figure 3a**); the median OS was higher in the DEB-TACE+ICIs than in the chemotherapy group: 13.2 months (95% CI: 4.977–21.423) versus 7.6 months (95% CI: 6.317–8.883)(*P*=0.015, **Figure 3b**).

### Prognostic Factors Analyses

The univariate and multivariate analyses results in the matched cohort were shown (**Table 3** **)**. Cox’s proportional hazard model suggested that the treatment option (DEB-TACE+ICIs vs. chemotherapy)(hazard ratio [HR]=2.325, 95% CI: 1.135–4.764, *P*=0.021), tumor size (≤5cm vs. >5cm)(HR=2.749, 95% CI: 1.185-6.378, *P*=0.019), and tumor number (single vs. multiple)(HR=1.721, 95% CI: 0.452-3.120, *P*=0.045), were independent predictive factor for PFS. Furthermore, treatment option (HR=2.882, 95% CI: 1.153–7.203, *P*=0.024), tumor size (HR=1.961, 95% CI: 1.124–3.321, *P*=0.023), and tumor number (HR=1.452, 95% CI: 0.567-2.148, *P*=0.032) were identified as the independent predictive factor for OS (**Table 3**).

**Table 3:** Prognostic factors associated with PFS and OS after PSM.

Variables	Progression-free survival						Overall survival					
	Univariate analysis			Multivariate analysis			Univariate analysis			Multivariate analysis		
	HR	95% CI	*P*	HR	95% CI	*P*	HR	95% CI	*P*	HR	95% CI	*P*
Age (years) ≤60/>60	1.559	0.768-3.166	0.219				1.866	0.798-4.364	0.150			
Sex Female/Male	0.686	0.339-1.388	0.295				0.911	0.401-2.070	0.823			
ECOG PS 0/1+2	1.149	0.549-2.404	0.712				1.862	0.725-4.785	0.196			
Child-Pugh class A5/A6+B7	0.809	0.468-2.645	0.809				2.021	0.775-5.273	0.150			
CA199 (u/ml) ≤37/>37	0.665	0.298-1.485	0.320				1.256	0.464-3.399	0.653			
CEA (ug/ml) ≤5/>5	1.202	0.566-2.556	0.632				1.495	0.582-3.840	0.404			
AST (U/L) ≤40/>40	1.003	0.458-2.196	0.994				1.145	0.484-2.709	0.759			
ALT (U/L) ≤35/>35	0.775	0.372-1.614	0.775				1.638	0.641-4.188	0.303			
Albumin level (g/L) ≤35/>35	0.337	0.188-1.770	0.337				0.278	0.110-1.112	0.216			
Tumor number Single/Multiple	1.379	0.678-1.805	0.035	1.721	0.452-3.120	0.045	1.204	0.525-1.762	0.061	1.452	0.567-2.148	0.032
Tumor size (cm) ≤5/>5	2.436	1.060-5.600	0.036	2.749	1.185-6.378	0.019	2.117	0.922-4.863	0.067	1.961	1.124-3.321	0.023
Tumor differentiation II/III+IV	1.189	0.531-2.662	0.674				1.623	0.551-4.781	0.379			
Extrahepatic metastasis Yes/No	0.841	0.376-1.883	0.674				0.616	0.209-1.814	0.379			
Treatment DEB-TACE+ICIs/Chemotherapy	2.170	1.020-4.619	0.044	2.481	1.150-5.354	0.021	2.906	1.174-7.194	0.006	2.882	1.153-7.203	0.024

Analyses were performed using Cox’s proportional hazards regression model. PFS, progression-free survival; OS, overall survival; PSM, propensity score matching; HR, Hazard Ratio; CI, confidence interval; ECOG PS, ALT, alanine transaminase; Eastern Cooperative Oncology Group performance score; TBIL, total bilirubin; AST, aspartate transaminase; DEB-TACE+ICIs, drug-eluting bead transarterial chemoembolization combined with immune checkpoint inhibitor.

### Safety

After PSM, the incidence of treatment-related AEs (TRAEs) in 2 groups was reported. TRAEs were observed at 92.5% (37/40), and no more than grade 4 occurred in the 2 groups (**Table 4**). ICIs-related AEs (irAEs) caused interruption of ICIs in 20% (4/20) of patients in the DEB-TACE+ICIs group. Moreover, TRAEs led to dose interruption and reduction of chemotherapy in 15.0% (3/20) and 15.0% (3/20) patients in the chemotherapy group, respectively.

**Table 4:** Summary of TRAEs after PSM.

Event, n (%)	Chemotherapy (n=20)			DEB-TACE+ICIs (n=20)				P			
Any TRAE	Any grade	Grade 1/2	Grade 3/4	Any grade		Grade 1/2	Grade 3/4	Any grade		Grade 1/2	Grade 3/4
	20(100.0)	17 (85.0)	8 (40.0)	17 (85.0)		16 (80.0)	6 (30.0)	0.072		0.677	0.507
**Hematologic toxic effects**											
Leukopenia Neutropenia Reduced hemoglobin level Thrombocytopenia	8 (40.0) 7 (35.0) 3 (15.0) 6 (30.0)	6 (30.0) 6 (30.0) 2 (10.0) 5 (25.0)	2 (10.0) 1 (5.0) 1 (5.0) 1 (5.0)		2 (10.0) 1 (5.0) 1 (5.0) 2 (10.0)	1 (5.0) 1 (5.0) 1 (5.0) 2 (10.0)	0 (0) 0 (0) 0 (0) 0 (0)		0.028 0.018 0.292 0.114	0.037 0.037 0.548 0.212	0.147 0.311 0.311 0.311
**Hepatic function**											
Increased AST Increased ALT	5 (25.0) 5 (25.0)	3(15.0) 3 (15.0)	2 (10.0) 2 (10.0)		9(45.0) 9 (45.0)	6 (30.0) 5 (25.0)	3(15.0) 4 (20.0)		0.185 0.185	0.256 0.429	0.633 0.376
Hyperbilirubinemia Hypoalbuminemia	4 (20.0) 4 (20.0)	4 (20.0) 4 (20.0)	0 (0) 0 (0)		6 (30.0) 5 (25.0)	6 (30.0) 5 (25.0)	0 (0) 0 (0)		0.465 0.705	0.465 0.705	>0.999 >0.999
**Nonhematologic toxic effects**											
Nausea Vomiting Anorexia Fatigue Constipation Abdominal pain Alopecia Rash Hypothyroidism RCCEP	8 (40.0) 9 (45.0) 4 (20.0) 7 (35.0) 1 (5.0) 3 (15.0) 3 (15.0) 1 (5.0) 0 (0) 0 (0)	8 (40) 7 (35.0) 4 (20.0) 4 (20.0) 1 (5.0) 3 (15.0) 2 (10.0) 1 (5.0) 0 (0) 0 (0)	0 (0) 2(10.0) 0 (0) 3 (15.0) 0 (0) 0 (0) 1 (5.0) 0 (0) 0 (0) 0 (0)		6 (30.0) 8 (40.0) 2 (10.0) 8 (40.0) 2 (10.0) 6 (30.0) 2 (10.0) 3 (15.0) 5 (25.0) 5 (25.0)	6 (30.0) 6 (30.0) 2 (10.0) 5 (25.0) 2 (10.0) 4 (20.0) 2 (10.0) 3 (15.0) 5 (25.0) 5 (25.0)	0 (0) 2 (10.0) 0 (0) 3 (15.0) 0 (0) 2 (10.0) 0 (0) 0 (0) 0 (0) 0 (0)		0.507 0.749 0.376 0.744 0.548 0.256 0.633 0.292 0.017 0.017	0.507 0.736 0.376 0.705 0.548 0.677 >0.999 0.292 0.017 0.017	>0.999 >0.999 >0.999 >0.999 >0.999 0.147 0.311 >0.999 >0.999 >0.999

TRAEs, treatment-related adverse events; PSM, propensity score matching; TRAE, treatment-related adverse event; DEB-TACE+ICIs, drug-eluting bead transarterial chemoembolization combined with immune checkpoint inhibitors; AST, aspartate aminotransferase; ALT, alanine transaminase; RCCEP, reactive cutaneous capillary endothelial proliferation.

The frequency of TRAEs related to hematologic toxic effects, including leukopenia (10.0% vs. 40.0%, *P*=0.028), and neutropenia (5.0% vs. 35.0%, *P*=0.018), were lower in DEB-TACE group than chemotherapy group. TRAEs related to hepatic function, including increased ALT and AST, hyperbilirubinemia, and hypoalbuminemia were no significant difference between the 2 groups (respectively, *P*>0.05). ICIs-related AEs (irAEs) mainly included hypothyroidism and reactive cutaneous capillary endothelial proliferation (RCCEP). The incidence rate of hypothyroidism was 25% (5/20), and RCCEP was 25% (5/20).

## DISCUSSION

This study suggested that DEB-TACE+ICIs improved survival in unresectable iCCA patients compared to chemotherapy. The result of median OS was increased from 6.6 months to 13.2 months, which might be related to the better ORR and PFS in patients who received DEB-TACE+ICIs compared to those treated with chemotherapy. Furthermore, multivariate analyses showed that DEB-TACE+ICIs was an independent predictor for prolonged PFS and OS. Thus, DEB-TACE+ICIs may be a good choice for unresectable iCCA who refuse systemic chemotherapy.

Previous studies (14, 23–25) have assessed unresectable iCCA patients treated with DEB-TACE and reported the median PFS was 3.0-3.9 months and OS was 10.5-12.4 months. Other studies reported that the median PFS and OS was 1.4-4.0 months and 4.3-12.7 months, respectively, in advanced biliary tract cancer patients treated with ICIs alone (26–29). Moreover, Chen et al. recently reported that the median PFS and OS was 6.1 months and 11.8 months, respectively, in advanced biliary tract cancer patients treated with camrelizumab plus Gemox (18). In current study, the median PFS and OS was 7.2 months and 13.2 months, respectively, in unresectable iCCA patients treated with DEB-TACE plus ICIs, which showed that DEB-TACE+ICIs might be an appropriate treatment plan in patients with unresectable iCCA. DEB-TACE is based on the administration of drug-eluting beads intra-arterially *via* catheter, which leads to local tumor necrosis, subsequently eliciting an anti-cancer immune response that may be further boosted with ICIs (11, 20). Liao et al. examined the effect of DEB-TACE on cellular immune function and regulatory T cells in patients with HCC and found that DEB-TACE can stimulate the cytokine spectrum and increase CD4^+^ and CD8^+^ T cells in PBMCs of HCC patients while reducing the Treg cell population (30). Moreover, Lee et al. found that DEB-TACE can change the Th1/Th2 balance in the tumor microenvironment (TME) in patients with HCC, thus improving survival (31). To sum up, DEB-TACE+ICIs may produce synergistic antitumor activity and contribute to improved survival.

In this study, the presence of tumor size >5cm or multiple tumors was identified as an independent risk factor for PFS and OS, which was consistent with previous studies (2, 14, 15, 32). DEB-TACE can enhance intratumoral concentration and release loaded chemotherapeutic agents in a controlled manner, further enhancing necrosis and leading to increase tumor response in iCCA patients (16). In addition, multiple tumors are easier to embolize by DEB-TACE, which results in a favorable prognosis of iCCA patients (16).

There were no new or unexpected TRAEs observed in current study. All the TRAEs were well-tolerated and consistent with previous reports (7, 15, 18, 19). The incidence of chemotherapy-related AEs (hematologic toxic effects) was higher in the chemotherapy group than in the DEB-TACE+ICIs group. The irAEs showed that RCCEP (25.0%) was lower than the result in a previous study (62%) (18), and hypothyroidism was consistent with a previous study (33). After receiving thyroxine or glucocorticoid, the irAEs were recovered within 2 weeks. These results suggested that DEB-TACE+ICIs did not increase the risk of TRAEs over chemotherapy, which showed that DEB-TACE+ICIs was safe.

There were some limitations in current study. Firstly, current study was a retrospective analysis, which might be subject to the impact of selection biases. We implemented the PSM model to resolve the effect result in confounding factors. A randomized clinical trial is required to validate the findings from this study. Secondly, these variables (including subgroup analysis) were not analyzed in current study due to the small sample size. Finally, we did not evaluate programmed cell death-Ligand 1 (PD-L1), mismatch repair protein (MMR) deficiency, and microsatellite instability-high (MSI-H) status before using ICIs.

In conclusion, DEB-TACE plus ICIs improved PFS and OS compared to chemotherapy with well tolerated in patients with unresectable iCCA.

## Data Availability Statement

The raw data supporting the conclusions of this article will be made available by the authors without undue reservation.

## Ethics Statement

The studies involving human participants were reviewed and approved by Sichuan Cancer Hospital. Written informed consent for participation was not required for this study in accordance with the national legislation and the institutional requirements. Written informed consent was not obtained from the individual(s) for the publication of any potentially identifiable images or data included in this article.

## Author Contributions

Conception and design: Huang X-Q and Xu G-H. Collection and assembly of data: Yang X-G, Sun Y-Y, and Li D-S. Data analysis and interpretation: Yang X-G. Manuscript writing: All authors. All authors contributed to the article and approved the submitted version.

## Funding

This study was supported by the Wu Jieping Medical Fund (NO.320.6750.2020-10-122), Beijing Medical Award Fund (NO.YXJL-2020-0972-0424), and a Special Research Fund Project of Tumour Interventional (NO.2020S04).

## Conflict of Interest

The authors declare that the research was conducted in the absence of any commercial or financial relationships that could be construed as a potential conflict of interest.

## Publisher’s Note

All claims expressed in this article are solely those of the authors and do not necessarily represent those of their affiliated organizations, or those of the publisher, the editors and the reviewers. Any product that may be evaluated in this article, or claim that may be made by its manufacturer, is not guaranteed or endorsed by the publisher.
